# Brain Structures and Cognitive Abilities Important for the Self-Monitoring of Speech Errors

**DOI:** 10.1162/nol_a_00015

**Published:** 2020-08-01

**Authors:** Ayan S. Mandal, Mackenzie E. Fama, Laura M. Skipper-Kallal, Andrew T. DeMarco, Elizabeth H. Lacey, Peter E. Turkeltaub

**Affiliations:** University of Cambridge, Department of Psychiatry, Cambridge, UK; Georgetown University Medical Center, Center for Brain Plasticity and Recovery and Department of Neurology, Washington, DC; Georgetown University Medical Center, Center for Brain Plasticity and Recovery and Department of Neurology, Washington, DC; Towson University, Department of Audiology, Speech-Language Pathology, and Deaf Studies, Towson, MD; Georgetown University Medical Center, Center for Brain Plasticity and Recovery and Department of Neurology, Washington, DC; Georgetown University Medical Center, Center for Brain Plasticity and Recovery and Department of Neurology, Washington, DC; Georgetown University Medical Center, Center for Brain Plasticity and Recovery and Department of Neurology, Washington, DC; MedStar National Rehabilitation Hospital, Research Division, Washington, DC; Georgetown University Medical Center, Center for Brain Plasticity and Recovery and Department of Neurology, Washington, DC; MedStar National Rehabilitation Hospital, Research Division, Washington, DC

**Keywords:** aphasia, self-monitoring, speech production, conflict monitoring, executive function, frontal white matter

## Abstract

The brain structures and cognitive abilities necessary for successful monitoring of one’s own speech errors remain unknown. We aimed to inform self-monitoring models by examining the neural and behavioral correlates of phonological and semantic error detection in individuals with post-stroke aphasia. First, we determined whether detection related to other abilities proposed to contribute to monitoring according to various theories, including naming ability, fluency, word-level auditory comprehension, sentence-level auditory comprehension, and executive function. Regression analyses revealed that fluency and executive scores were independent predictors of phonological error detection, while a measure of word-level comprehension related to semantic error detection. Next, we used multivariate lesion-symptom mapping to determine lesion locations associated with reduced error detection. Reduced overall error detection related to damage to a region of frontal white matter extending into dorsolateral prefrontal cortex. Detection of phonological errors related to damage to the same areas, but the lesion-behavior association was stronger, suggesting that the localization for overall error detection was driven primarily by phonological error detection. These findings demonstrate that monitoring of different error types relies on distinct cognitive functions, and provide causal evidence for the importance of frontal white matter tracts and the dorsolateral prefrontal cortex for self-monitoring of speech.

## INTRODUCTION

Although fluent speech is littered with errors, healthy speakers can identify and repair these mistakes. Successful communication depends on this ability to self-correct. Previous studies of error detection have shown that it predicts positive therapeutic outcomes for both production and comprehension in aphasia (Marshall, Neuburger, & Phillips, 1994). Schwartz, Middleton, Brecher, Gagliardi, and Garvey (2016) provided evidence that the correction of semantic errors promotes an adaptive change that allows aphasic patients to learn from their mistakes. These results imply that self-monitoring has important consequences for aphasia recovery.

Despite the evidence that self-monitoring plays a role in language relearning, little is known about the processes underlying it. There are currently two broad categories of theories regarding self-monitoring. The first category consists of comprehension-based models, where persons detect errors by listening to their own speech (Postma, 2000). When speakers hear themselves say something different from what was intended, then they can identify and correct the error. Chief among comprehension-based models for error detection is the perceptual loop model initially posed by Levelt (1983). Under the perceptual loop model, an outer auditory loop monitors overt speech using speech comprehension systems. Pre-articulatory inner speech is monitored as well via an inner loop that also relies on speech comprehension (Indefrey & Levelt, 2004). This model is parsimonious because it does not assume the existence of a system dedicated to self-detecting errors. Rather, it suggests that the same mechanism that allows people to comprehend the speech of others also allows them to detect their own errors.

Comprehension-based models for self-monitoring predict that poor error detection will correlate with poor comprehension abilities. If one detects an error by comprehending one’s own overt speech, then a person who has difficulties comprehending the speech of others should also have difficulties with self-monitoring. However, Nickels and Howard (1995) found no correlation between error detection and any of three measures of auditory comprehension. Furthermore, comprehension and error detection doubly dissociate: There have been case studies of patients with aphasia who accurately detect their own errors but demonstrate poor comprehension (Marshall, Rappaport, & Garcia-Bunuel, 1985), as well as patients with poor error detection yet intact comprehension (Butterworth & Howard, 1987; Liss, 1998; Marshall, Robson, Pring, & Chiat, 1998). It is worth noting, however, that while these studies provide substantial evidence against an overt-speech monitoring loop, it has been more difficult to test the functioning of an inner-speech monitoring loop.

In contrast to comprehension-based speech monitors, some authors have proposed production-based self-monitoring systems, where information from the speech production process itself can be used to detect an error. Particularly notable amongst production-based models for error detection is the conflict-based monitor proposed by Nozari, Dell, and Schwartz (2011). In Nozari et al.’s model, the language system emits conflict signals that arise from competition between various semantic features or phonological units activated during naming. These signals are received by the anterior cingulate cortex, which processes the conflict and alerts the lateral prefrontal cortex (LPFC) to exert top-down control to resolve conflict (Botvinick, Braver, Barch, Carter, & Cohen, 2001; Yeung, 2015; Yeung, Botvinick, & Cohen, 2004). The conflict-based model has recently gained support from a behavioral study in neurotypical children (Hanley, Cortis, Budd, & Nozari, 2016) and a functional MRI study in healthy adults (Gauvin, De Baene, Brass, & Hartsuiker, 2015).

The self-monitoring models mentioned above make different predictions regarding the neural and behavioral correlates of error detection. The perceptual loop model predicts that detection should depend on comprehension. Consequently, the perceptual loop model also predicts detection to rely on brain structures that subserve comprehension, that is, primarily regions within the temporal lobe (Hillis, Rorden, & Fridriksson, 2017). Production monitors in general predict that lesions to areas important for word production, broadly within the frontal lobe, should impair error detection capabilities (Catani et al., 2013; Mandelli et al., 2014). The conflict-based model in particular also predicts that regions involved in domain-general cognitive processing, such as the anterior cingulate cortex (ACC) or LPFC, should also be involved (Yeung, 2015).

People with aphasia sometimes exhibit impairments in monitoring that are specific to one type of error over another, such as detecting each of their phonological errors but failing to notice any of their semantic errors (Marshall et al., 1985; Stark, 1988). Such observations suggest that different cognitive processes may be involved in the monitoring of different types of errors. This notion has received empirical support in recent years from studies that have found differences in the timing of and the learning from semantic versus phonological error monitoring (Schuchard, Middleton, & Schwartz, 2017; Schwartz et al., 2016). Comprehension-based models do not explicitly account for differential monitoring of phonological versus semantic errors, although one may expect a selective deficit in the monitoring of one type of error if the comprehension system has incurred a selective impairment in either phonological or semantic processing. However, a case study of a patient with impaired phonological auditory processing (in the form of auditory agnosia) yet preserved reading comprehension demonstrated the opposite of this expectation: The subject detected almost all of her phonological errors but ignored her semantic errors (Marshall et al., 1985). Production-based monitors for error detection have been proposed for virtually every stage of word production, from lemma selection to tactile feedback following word articulation (Postma, 2000), and therefore could support differential monitoring of errors that arise at different stages. Nozari et al. (2011) found that measures of the lexical-semantic and lexical-phonological stages of naming predicted detection of semantic and phonological errors, respectively. The authors suggested that damage to the lexical-semantic or lexical-phonologic stages of naming causes noise to predominate in the activations of representations by these systems, which obscures the ability of a monitor to detect conflict related to errors.

An examination of the brain structures and cognitive abilities associated with detection of different types of naming errors could help not only distinguish between current self-monitoring accounts but also extend existing theories into new domains.

### The Current Study

In the present study, we tested the anatomical and behavioral predictions of the perceptual loop and conflict-based models for error detection in a group of participants with post-stroke aphasia. First, we determined whether error detection within the context of a picture-naming task related to cognitive abilities proposed to contribute to monitoring, including naming ability, fluency, word-level auditory comprehension, sentence-level auditory comprehension, and executive function. Then, we used support vector regression lesion-symptom mapping (SVR-LSM) to map the brain areas necessary for error detection, and probed the interrelationships between the behavioral and neural correlates of detection ability.

## MATERIALS AND METHODS

### Participants

Data for the current study were pooled from cohorts of left hemisphere stroke survivors recruited for two different studies at Georgetown University and MedStar National Rehabilitation Hospital. Forty-nine patients (Cohort 1) were participating in a battery of tasks to determine baseline language abilities in a transcranial direct current stimulation clinical trial. Fifty-four patients (Cohort 2) were participating in a study designed to probe the subjective experience of inner speech in aphasia. Twenty-three patients participated in both studies, yielding a total of 80 potential participants after combining the two cohorts. All participants were native English speakers, had no history of other brain disorder or damage, had anterior circulation strokes, and were tested at least six months after the stroke. Participants were excluded from analyses if they produced too few errors of the type being examined to assess error detection (see Dependent Variables below). Demographic details of the participants included in each analysis are listed in Table 1. The study was approved by the Georgetown University Institutional Review Board, and written informed consent was obtained from all study participants prior to enrollment in the study.

**Table 1.** Demographic data and performance on measures of interest for study participants

	Total error detection group (*N* = 64)	Phonological error detection group (*N* = 58)	Semantic error detection group (*N* = 32)
Age (years)	60.4 (9.3)	60.0 (9.2)	60.6 (8.4)
Sex (male/female)	42/22	38/20	19/13
Time since stroke (months)	47.6 (48.8)	43.4 (44.6)	49.3 (57.2)
Education (years)	16.0 (2.9)	15.9 (3.0)	16.3 (2.6)
Handedness (left/right/ambidextrous)	56/5/3	51/4/3	27/4/3
Lesion size (cm^3^)	117.9 (81.0)	117.8 (81.4)	121.6 (80.5)
Naming accuracy	0.52 (0.31)	0.51 (0.31)	0.48 (0.29)
Phonological errors	0.48 (0.21)	0.51 (0.19)	0.39 (0.19)
Semantic errors	0.16 (0.12)	0.15 (0.10)	0.24 (0.11)
Total error detection	0.40 (0.24)	0.40 (0.25)	0.42 (0.21)
Phonological error detection	0.40 (0.28)	0.39 (0.28)	0.42 (0.24)
Semantic error detection	0.38 (0.25)	0.38 (0.25)	0.38 (0.25)
WAB-R auditory comprehension	0.93 (0.065)	0.92 (0.065)	0.92 (0.074)
Word-to-picture matching	0.92 (0.11)	0.91 (0.11)	0.92 (0.10)
MLU	4.2 (2.3)	4.1 (2.3)	4.4 (2.2)
Digit span forwards	4.7 (3.2)	4.5 (3.2)	5.3 (3.6)
Digit span backwards	2.2 (2.0)	2.0 (1.9)	2.3 (2.2)
Digit span difference	2.5 (2.4)	2.5 (2.4)	3.0 (2.3)
Spatial span forwards	6.3 (2.2)	6.3 (2.2)	6.1 (2.5)
Spatial span backwards	5.1 (2.4)	5.3 (2.3)	5.3 (2.4)
Spatial span difference	1.1 (1.7)	0.95 (1.7)	0.81 (1.4)

*Note*. Standard deviations are presented in parentheses. Each behavioral measure has been divided by its maximum possible score, except MLU, digit span, and spatial span tasks, which have no maximum score. Phonological error detection and semantic error detection groups are subsets of the total error detection group. Twenty-nine participants are in both the phonological error detection and semantic error detection groups. WAB-R = Western Aphasia Battery-Revised, MLU = mean length of utterance.

### Behavioral Tasks

All relevant tasks that had been administered to both Cohort 1 and Cohort 2 were selected for the study. These included tasks that measure confrontation picture-naming performance and error detection, as well as several functions proposed to be important for error monitoring, including word-level auditory comprehension, sentence-level speech comprehension, short-term memory, working memory, executive function, and fluency. In participants for whom scores were available from both prior studies, the scores of the two equivalent tests were averaged.

#### Philadelphia Naming Test

Participants from Cohort 1 were administered a 60-item version of the Philadelphia Naming Test (PNT; Roach et al., 1996). The PNT is a picture-naming task where participants must name a series of black and white drawings. Participants from Cohort 2 were administered a 120-item naming task, which included the 60-item PNT plus an additional 60 items. For patients who participated in both prior studies, only the 60 PNT items that matched across both groups were used to provide an overall naming accuracy score, but all available trials were pooled across both tasks for the purpose of coding error types and error detection. To determine whether this pooling substantially impacted the results, analyses were also conducted using the error detection performance from only the 60-item PNT administered to both cohorts (see the online supporting information located at https://www.mitpressjournals.org/doi/suppl/10.1162/nol_a_00015).

#### Western Aphasia Battery-Revised auditory comprehension

The Western Aphasia Battery-Revised (WAB-R) Yes/No Questions task was administered. This task requires a yes/no response to 20 items including questions that are biographical, environmental, and noncontextual/grammatically complex in nature (Kertesz, 2007) and assesses sentence-level comprehension ability.

#### Digit span forwards

Participants were asked to repeat strings of numbers of increasing length in the same order in which they heard the sequence. Two strings were presented at each length, and testing stopped after both strings at a given length were recited incorrectly. The total number of strings recited correctly was taken, in which a low number indicates poor verbal short-term memory.

#### Word-to-picture matching

Participants heard a word and pointed to the target item in a field of semantically related pictures. The version of the task used in Cohort 1 included a field of six pictures, whereas in Cohort 2 the field included four pictures. Both tasks included 48 trials. Accuracies on the two tasks were not different for the 23 patients who performed both, paired *t*(22) = 0.776, *p* = 0.45, so the tasks were treated as equivalent. Poor performance on this task is interpreted to indicate word-level comprehension impairment, but could reflect other factors (see Discussion below).

#### Mean length of utterance

Mean length of utterance (MLU) during a picture description task was used as a measure of speech fluency. This quantity is derived by calculating the mean number of words used in each utterance. Participants from Cohort 1 described the picnic scene from the WAB–R, while those from Cohort 2 described the “Cookie Theft” picture from the Boston Diagnostic Aphasia Examination (Goodglass, Kaplan & Barresi, 2001). The scores from the two pictures were not different for the patients for whom both scores were available, paired *t*(19) = 1.20, *p* = 0.244, so the tasks were treated as equivalent. Five subjects were missing data and so are excluded from analyses using MLU.

#### Digit span backwards

The same procedures were followed as for digit span forwards, except that participants were asked to recite the number strings in reverse order, a manipulation that is typically thought to tax executive control of working memory.

#### Digit span difference

The difference between the forwards and backwards digit span scores was calculated. A high number on the measure is interpreted here to indicate poor executive control, but could also reflect other factors (see Discussion).

#### Spatial span forwards

The Corsi-Block tapping task was used (Corsi, 1972). Participants were asked to tap a sequence of blocks of increasing length in the same order in which they saw an examiner tap the blocks. Two sequences were presented at each length, and testing stopped after both sequences of a given length were repeated incorrectly. The total number of sequences repeated correctly was taken, in which a low number indicates poor nonverbal short-term memory.

#### Spatial span backwards

The same procedures were followed as for spatial span forwards, except that participants were asked to tap the sequence of blocks in reverse order, a manipulation that is typically thought to tax executive control of working memory.

#### Spatial span difference

The difference between the forwards and backwards spatial span scores was calculated. A high number on this measure is interpreted to indicate poor executive control, but could also reflect other factors (see Discussion).

### Coding Naming Responses for Error Type and Error Detection

#### Error type

Videos of naming responses were transcribed into the International Phonetic Alphabet and scored offline for accuracy, error type, and error detection. Error coding was based on the PNT rules (Roach et al., 1996). Only the first naming attempt for each item was coded. False starts and fragments were not considered as first naming attempts as per PNT scoring rules. Errors, whether words or nonwords, were coded as phonological if they shared the stressed vowel, at least two phonemes, or the first or last phoneme as the target. Errors that were semantically related to the target were coded as semantic errors. Errors that were both phonologically and semantically related to the target were coded with mixed errors and were thus considered as neither phonological nor semantic. The other error types specified in the PNT scoring rules were coded but are not considered here. Seventeen subjects (four of whom received the 120-item naming task) were graded by two independent scorers to determine interrater reliability. Errors received the same error code from different scorers on 91.7% of trials. This is comparable to the interrater reliability observed in similar studies (Schwartz et al., 2016).

#### Error detection

Error detection was assessed within the context of the picture-naming task using an adapted version of the coding protocol developed by Schwartz and colleagues (Schwartz et al., 2016). A participant was said to have detected an error if they uttered a statement indicating awareness that an error was made or if they attempted to self-correct the error. In other words, when a participant made an error, it would count as detected if the person either followed the errant response with a statement like “no, not that,” or made an attempt to correct their first response (e.g., “cat … I mean dog”), indicating recognition that the first response was incorrect. Attempts at self-correction were coded as detected errors regardless of whether the second attempt was correct or incorrect because either provides evidence that the individual is aware of the error. Only the first naming attempt for each item was scored for error detection; detection or correction of errors in attempts at self-correction were not scored (e.g., for “cat … I mean dog … I mean bird” only the first self-correction is scored). This ensured that there was only one error detection score per item, which reduced bias toward individuals who made many attempts at self-correction, and made it simpler to interpret detection of specific error types. Nonverbal indicators of error acknowledgement, such as head shakes, were not coded. Repetition of the initial naming attempt (e.g., “cat … cat”) was also not coded as an error detection. The accuracy of attempts at self-correction was recorded, but those data are not considered here because there was not adequate power to further divide the types of detections and corrections. Consistent with protocols in prior studies (Nozari et al., 2011; Schuchard et al., 2017; Schwartz et al., 2016), participants were not given instructions to indicate awareness of an error or to self-correct, so all error detection was spontaneous.

### Dependent Variables

#### Total error detection score

The total error detection score was calculated by dividing the number of detections by the total number of errors on initial naming attempts, and so is expressed as the proportion of errors that were detected by each individual. Errors of all types—phonological, semantic, mixed, and so forth—were considered for this measure. Total error detection scores were not considered from participants who produced fewer than 10 errors, leaving 64 participants in the analyses of total error detection.

#### Error detection

The phonological error detection score was calculated by dividing the number of detections of phonological errors by the total number of phonological errors produced. Phonological error detection scores were not considered from participants who produced fewer than five phonological errors, leaving 58 participants in the analyses of phonological error detection.

The semantic error detection score was calculated by dividing the number of detections of semantic errors by the total number of semantic errors produced. Semantic error detection scores were not considered from participants who produced fewer than five semantic errors, leaving 32 participants in the analyses of semantic error detection.

### Behavioral Analysis

Statistical analyses were conducted in SPSS 25 (https://www.ibm.com/products/spss-statistics). Outlier scores (greater or less than three interquartile distances from the median) were excluded from analyses. This resulted in exclusion of two individuals’ word-picture matching scores.

A series of one-way analyses of variance (ANOVAs) was first used to confirm that scores of each test did not differ between the participants from only Cohort 1, only Cohort 2, and those who were in both cohorts.

Bivariate correlations were used to screen for interrelationships between variables of interest. Next, three multiple linear regression analyses were performed with backwards elimination of predictors with *p* > 0.1 to determine which behavioral scores related to detection of each error type (all errors, phonological errors, semantic errors). Only scores that were univariately correlated (uncorrected for multiple comparisons) with at least one of the dependent variables were entered into these regression analyses. These scores included word-to-picture matching, MLU, digit span difference, spatial span backwards, and spatial span difference.

To investigate whether the monitoring of different types of errors related to distinct cognitive abilities, we performed two additional regression analyses for phonological and semantic error detection, where detection of the other error type was included as predictor along with the original cognitive measures. These analyses were necessarily limited to individuals who were in both the phonological error detection and semantic error detection groups and had scores for all behavioral predictors (*N* = 27).

### Neuroimaging

#### MRI acquisition and preprocessing

Three-dimensional T1-weighted MRIs were acquired from participants on a 3.0 T Siemens Trio scanner with the following parameters: TR = 1,900 ms; TE = 2.56 ms; flip angle = 9°; 160 contiguous 1 mm sagittal slices; field of view (FOV) = 250 × 250 mm; matrix size = 246 × 256; voxel size 1 mm^3^. A T2-weighted sampling perfection with application optimized contrasts using a different flip angle evolution (SPACE) sequence was acquired with the following parameters: 176 sagittal slices; slice thickness = 1.25 mm; FOV = 240 × 240 mm; matrix size = 384 × 384; TR = 3,200 ms; echo train length = 145, variable TE; variable flip angle; voxel size = 0.625 × 0.625 × 1.25 mm^3^.

Lesions were manually traced on coregistered T1-weighted and T2-weighted images in native space using ITK-SNAP 3.6 (http://www.itksnap.org) by a board certified neurologist (P.E.T.). Native space MPRAGEs and lesion tracings were warped to Montreal Neurological Institute (MNI) space using the Clinical Toolbox Older Adult Template as the target template (Rorden, Bonilha, Fridriksson, Bender, & Karnath, 2012) via a custom pipeline. First, brain parenchyma was extracted from each native space image by applying a mask intended to minimize the clipping of gray matter edges. The initial mask was generated by combining the lesion tracing image (binarized) with white and gray matter tissue probability maps generated by the unified segmentation procedure in SPM12 (http://picsl.upenn.edu/software/ants/) applied to the original native space image, cost-function masked with the lesion tracing. The resulting mask was blurred and inverted to remove nonbrain tissue from the image.

The resulting brain extracted image was then normalized using Advanced Normalization Tools software (ANTs; http://picsl.upenn.edu/software/ants/; Avants, Tustison, & Song, 2009). Lesion masking was used at each step of the ANTs process. After bias field correction was applied, normalization proceeded using a typical ANTs procedure, including a rigid transform step, an affine transform step, and a nonlinear symmetric normalization (SyN) step. Next, the output of this initial ANTs warp was recursively submitted to three additional applications of the SyN step. Finally, the resulting linear (rigid and affine) and four nonlinear warp fields were concatenated, and the original native space MPRAGE and lesion tracings were transformed to the template space using BSpline interpolation. This iterative application of nonlinear warping was intended to improve normalization of expanded ventricles and displaced deep structures in individuals with large lesions. The normalized lesion tracings were finally downsampled to 2.5 mm^3^.

#### Lesion-symptom mapping

We implemented SVR-LSM (Zhang, Kimberg, Coslett, Schwartz, & Wang, 2014) using a MATLAB-based toolbox (DeMarco & Turkeltaub, 2018) running under MATLAB R2017a (The MathWorks, Inc.). SVR-LSM was used to identify the left hemisphere areas associated with impaired self-monitoring. SVR-LSM applies a machine learning based algorithm to find lesion-symptom relationships more sensitively and specifically than traditional mass-univariate lesion-symptom mapping approaches (Mah, Husain, Rees, & Nachev, 2014). Only voxels damaged in at least 10% of the participants in the study were considered for each analysis. Lesion volume confounds were controlled in all analyses by regressing the lesion volume out of both behavioral scores and lesion masks, a method that provides rigorous control of lesion volume and is more sensitive than alternative approaches (DeMarco & Turkeltaub, 2018). Voxel-wise beta-values were thresholded at *p* < 0.005 using 10,000 permutations of the behavioral scores to generate voxel-wise null distributions. To correct for multiple comparisons, a cluster threshold determined from the 10,000 permutation maps was applied to control the familywise error rate at 0.05 (Mirman et al., 2018). SVR-LSM analyses were performed examining lesion locations associated with failure to detect each error type (total errors, phonological errors, semantic errors). An additional SVR-LSM analysis for phonological error detection was performed where MLU was added as a covariate. MRIs were not obtained on some participants, so the sample sizes for the SVR-LSM analyses were *N* = 57 for total errors, *N* = 51 for phonological errors, and *N* = 29 for semantic errors.

Model quality was assessed in two ways. The first was prediction accuracy, which is a density of correlation coefficients between predicted scores and training scores across 10 replications of a 5-fold cross-validated model. The mean of this density (average correlation coefficient) is used to summarize how well the predicted scores trend with the real scores. However, previous work has observed that the quality of back-projected spatial patterns cannot be assessed on the basis of prediction accuracy alone (Rasmussen et al., 2012). Indeed, this work has observed a trade-off between model visualization reproducibility and prediction accuracy. Therefore, a second metric produced by Rasmussen et al. (2012), pattern reproducibility index, was used to assess reproducibility of the back-projected pattern. Pattern reproducibility index is calculated as a density of voxel-wise correlation coefficients computed pairwise between 10 replicates of SVR-β maps, each generated using a random 80% of observations. Model quality measures were as follows: total detection accuracy 0.31 (*SD* 0.07), reproducibility *r* = 0.88 (*SD* 0.05); phonological detection accuracy 0.25 (*SD* 0.07), reproducibility *r* = 0.89 (*SD* 0.04); phonological detection controlling MLU accuracy 0.26 (*SD* 0.11), reproducibility *r* = 0.86 (*SD* 0.04); semantic detection accuracy 0.36 (*SD* 0.15), reproducibility *r* = 0.75 (*SD* 0.12).

#### Region of interest analyses for semantic error detection

To compensate for the smaller sample size of the semantic error detection SVR-LSM analyses, we selected theory-driven regions of interest (ROIs) from the Harvard Oxford Cortical atlas (https://fsl.fmrib.ox.ac.uk/fsl/fslwiki/Atlases) to determine whether brain regions important for auditory comprehension or speech production related to semantic error monitoring. Selected ROIs included the posterior superior temporal gyrus, the anterior superior temporal gyrus, the posterior middle temporal gyrus, Heschl’s gyrus, the planum temporale, the angular gyrus, the inferior frontal gyrus (IFG), the pars triangularis, and the middle frontal gyrus. Lesion load for each ROI was calculated for each participant by dividing the number of voxels that overlapped between the lesion mask and the ROI by the total number of voxels within the ROI. Partial correlations were calculated between ROI lesion load and semantic error detection, controlling for total lesion volume.

#### Data availability

The data that support the findings of this study are available on request from the corresponding author. The data are not publicly available due to their containing information that could compromise the privacy of research participants. Source code that was used to conduct the SVR-LSM analyses in this study is available at https://github.com/atdemarco/svrlsmgui/.

## RESULTS

### Relationships of Behavioral Scores to Error Detection

Average scores across the groups are shown in Table 1. Since the data were derived from two partially overlapping patient cohorts, we first performed a series of one-way ANOVAs to confirm that cohort differences did not introduce biases in the variables of interest (age, time since stroke, education, PNT, word-to-picture matching, WAB-R auditory comprehension, MLU, digit span forwards, digit span backwards, digit span difference, spatial span forwards, spatial span backwards, spatial span difference, total error detection, phonological error detection, semantic error detection). No effects of cohort (participants from only cohort 1 vs. participants from only cohort 2 vs. individuals from both cohorts) were identified (all *p* > 0.05).

Bivariate correlations between behavioral variables of interest and each dependent variable are provided in Table 2 and Table 3. Intercorrelations among behavioral variables of interest are provided in Table 4. Phonological error detection and semantic error detection were correlated with one another (*r* = 0.570; *p* = 0.0012; *N* = 29), and this relationship persisted after controlling for lesion volume (*r* = 0.502; *p* = 0.011; *N* = 23). Regression models were used to identify predictors of error detection, first examining detection of all errors, and then semantic and phonological errors separately. Only behavioral measures that were univariately correlated with at least one of the dependent variables were entered into these regression models. These measures were word-to-picture matching, MLU, digit span difference, spatial span backwards, and spatial span difference. Total error detection was predicted by MLU (standardized β = 0.380; *p* = 0.002; variance inflation factor [VIF] = 1.025) and spatial span difference (standardized β = −0.333; *p* = 0.006; VIF = 1.025) with an overall adjusted *R*^2^ of 0.268, *F*(2, 54) = 11.3, *p* < 0.001. Phonological error detection was predicted by MLU (standardized β = 0.428; *p* = 0.001; VIF = 1.031), digit span difference (standardized β = −0.249; *p* = 0.04; VIF = 1.061), and spatial span difference (standardized β = −0.255; *p* = 0.038; VIF = 1.091) with an overall adjusted *R*^2^ of 0.334, *F*(3, 48) = 9.54; *p* < 0.001. Semantic error detection was predicted only by word-to-picture matching (standardized β = 0.516; *p* = 0.003; VIF = 1.000) with an overall adjusted *R*^2^ of 0.240, *F*(1, 28) = 10.173, *p* = 0.003. These results were mostly preserved when reanalyzed using error detection performance based only on the 60 PNT items administered to all participants (see the online supporting information for this article).

**Table 2.** Correlations between error detection and demographic variables

	Total error detection (*N* = 64)	Phonological error detection (*N* = 58)	Semantic error detection (*N* = 32)
Age	0.269*	0.200	−0.183

Time since stroke	−0.035	−0.044	0.080

Education	0.211	0.101	−0.001

*Note*. ***p < 0.05, after Bonferroni correction for 9 comparisons, **p < 0.01 uncorrected, *p < 0.05 uncorrected.

**Table 3.** Correlations between error detection and other variables

	Total error detection	Phonological error detection	Semantic error detection
PNT	0.027 (*N* = 64)	−0.063 (*N* = 58)	0.079 (*N* = 32)
WAB-R auditory comprehension	0.115 (*N* = 62)	0.022 (*N* = 56)	0.139 (*N* = 32)
Word-to-picture matching	0.302* (*N* = 62)	0.244 (*N* = 56)	0.523** (*N* = 31)
Mean length of utterance	0.514*** (*N* = 59)	0.502*** (*N* = 54)	0.365 (*N* = 31)
Digit span forwards	−0.074 (*N* = 59)	−0.210 (*N* = 54)	0.033 (*N* = 31)
Digit span backwards	0.148 (*N* = 59)	0.084 (*N* = 54)	0.082 (*N* = 31)
Digit span difference	−0.226 (*N* = 59)	−0.340** (*N* = 54)	−0.023 (*N* = 31)
Spatial span forwards	0.096 (*N* = 59)	0.022 (*N* = 54)	0.265 (*N* = 31)
Spatial span backwards	0.327** (*N* = 59)	0.276* (*N* = 54)	0.377* (*N* = 31)
Spatial span difference	−0.329** (*N* = 59)	−0.352* (*N* = 54)	−0.171 (*N* = 31)

*Note*. Sample sizes differ due to missing scores. WAB-R = Western Aphasia Battery-Revised. ***p < 0.05 after Bonferroni correction for 33 comparisons, **p < 0.01 uncorrected, *p < 0.05 uncorrected.

**Table 4.** Correlations between behavioral measures

	WAB-R Yes/No	Word-to-picture matching	MLU	Digit span fwd	Digit span bwd	Digit span diff	Spatial span fwd	Spatial span bwd	Spatial span diff
PNT	0.277*	0.679***	0.491***	0.562***	0.529***	0.313*	0.314*	0.253*	0.055
WAB-R Yes/No		0.337**	0.332*	0.471***	0.304*	0.374**	0.140	0.032	0.137
Word-to-picture matching			0.456***	0.340**	0.440***	0.079	0.401***	0.416***	−0.069
MLU				0.474***	0.626***	0.092	0.190	0.252	−0.104
Digit span fwd					0.680***	0.780***	0.379**	0.261*	0.128
Digit span bwd						0.071	0.474***	0.490***	−0.065
Digit span diff							0.110	−0.063	0.230
Spatial span fwd								0.723***	0.292*
Spatial span bwd									−0.450***

*Note*. Correlations are performed for the full sample (*N* = 64). Sample size varies between 57 and 64 on individual correlations because of missing values on some tests. PNT = Philadelphia Naming Test, WAB-R = Western Aphasia Battery-Revised, MLU = mean length of utterance, fwd = forwards, bwd = backwards, diff = difference. ***p < 0.05 after Bonferroni correction for 33 comparisons, **p < 0.01 uncorrected, *p < 0.05 uncorrected.

To determine whether the behavioral predictors of phonological and semantic error detection were specific to the monitoring of those respective error types, we performed two additional regression analyses for phonological and semantic error detection, where detection of the other error type was included as a predictor along with the original cognitive measures. Phonological error detection was predicted by semantic error detection (standardized β = 0.587; *p* = 0.001; VIF = 1.23), spatial span difference (standardized β = −0.321; *p* = 0.039; VIF = 1.02), spatial span backwards (standardized β = −0.336; *p* = 0.047; VIF = 1.23), and MLU (standardized β = 0.283; *p* = 0.071; VIF = 1.08) with an overall adjusted *R*^2^ of 0.461, *F*(4, 22) = 6.56; *p* = 0.001. Semantic error detection was predicted by phonological error detection (standardized β = 0.392; *p* = 0.018; VIF = 1.14), and word-to-picture matching (standardized β = 0.464; *p* = 0.006; VIF = 1.14) with an overall adjusted *R*^2^ of 0.455, *F*(2, 24) = 11.8; *p* < 0.001.

### Lesion-Symptom Mapping

**Figure 1.**
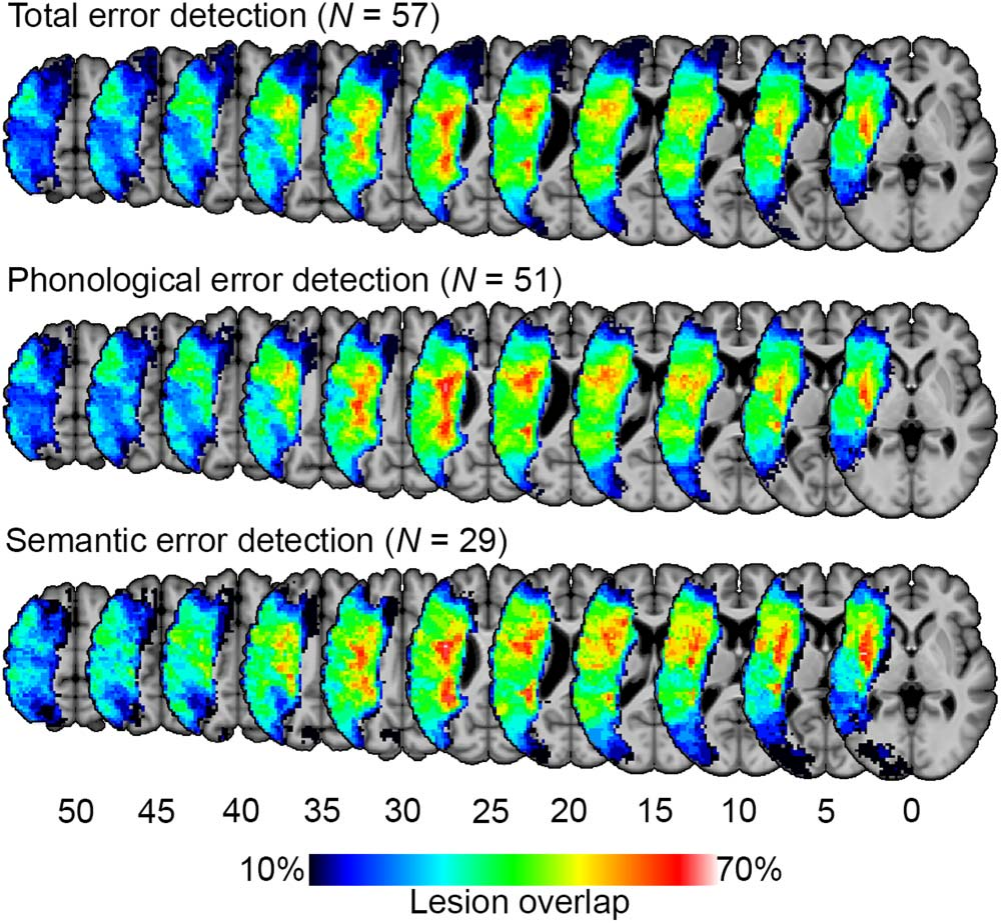
Lesion overlap maps for study participants. Lesion-symptom mapping analyses were limited to voxels that were lesioned in at least 10% of participants (i.e., at least six participants for total and phonological error detection, at least three for semantic error detection).

**Figure 2.**
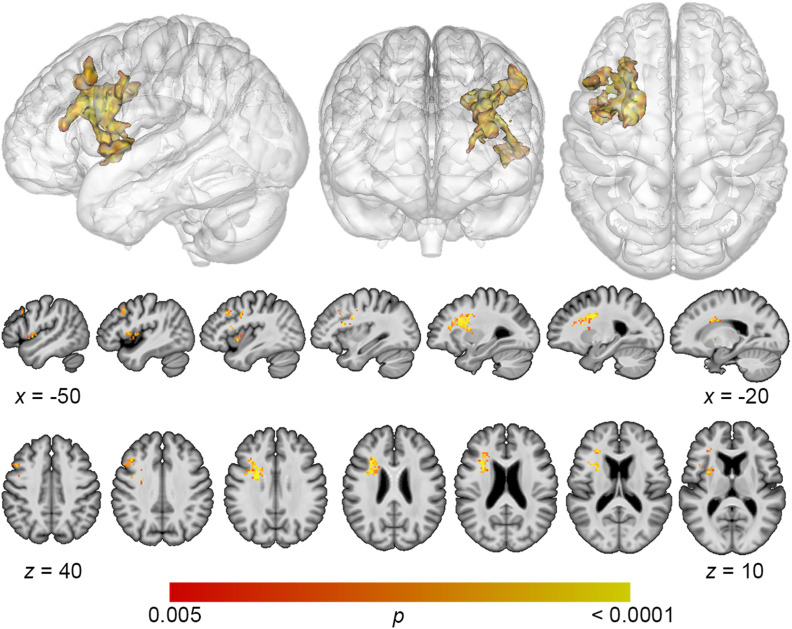
Multivariate lesion-symptom mapping results for total error detection. Support vector regression lesion-symptom mapping analysis demonstrated that decreased detection of all error types was associated with damage to a large region of frontal white matter and dorsolateral prefrontal cortex (voxel-wise *p* < 0.005, cluster-level familywise error < 0.05).

**Figure 3.**
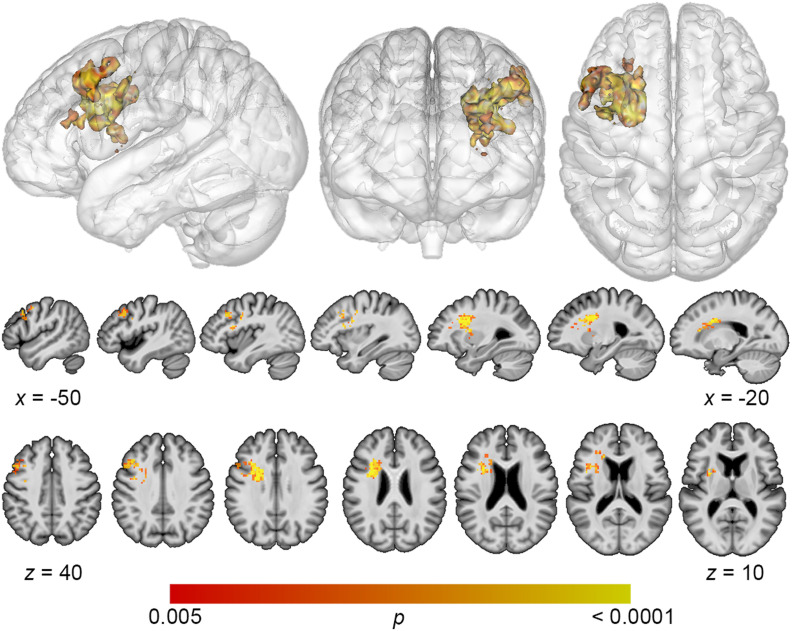
Multivariate lesion-symptom mapping results for phonological error detection. Reduced detection of phonological errors related to damage to frontal white matter and dorsolateral prefrontal cortex (voxel-wise *p* < 0.005, cluster-level familywise error < 0.05).

**Figure 4.**
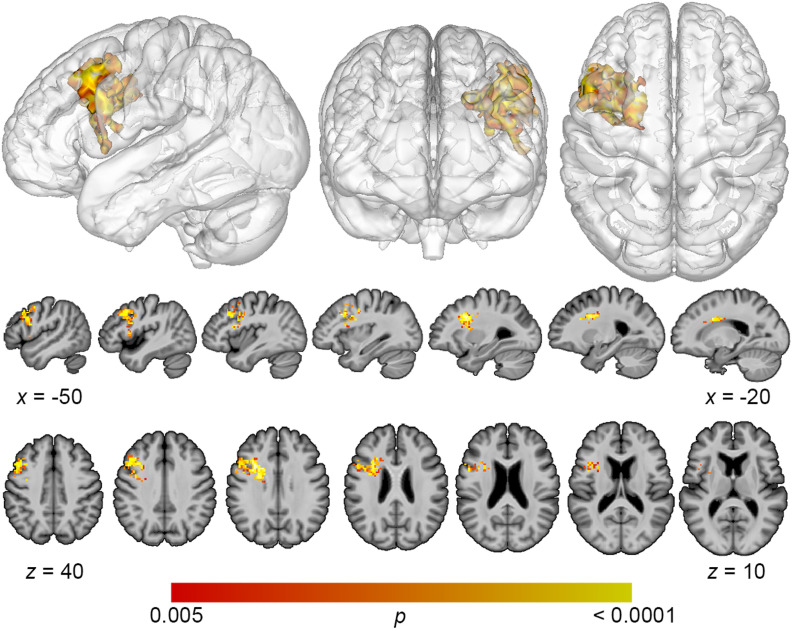
Multivariate lesion-symptom mapping results for phonological error detection, controlling for fluency. After adding mean length of utterance as a covariate, reduced detection of phonological errors related to damage to dorsolateral prefrontal cortex (voxel-wise *p* < 0.005, cluster-level familywise error < 0.05).

Lesion overlap maps demonstrated good coverage of the left middle cerebral artery territory for all analyses (Figure 1). Lesion volume correlated modestly with total error detection (*r* = −0.278; *p* = 0.04), and a trending relationship was observed with phonological error detection (*r* = −0.243; *p* = 0.09) and semantic error detection (*r* = −0.31; *p* = 0.10). Lesion volume was subsequently controlled for in all SVR-LSM analyses. SVR-LSM analyses demonstrated that decreased total error detection mapped onto lesions in a large region of frontal white matter and the dorsolateral prefrontal cortex (DLPFC) (Figure 2). A single cluster was identified with a volume of 7,156 mm^3^, with center of mass at MNI coordinates −32.7, 8.7, 20.5. Decreased detection of phonological errors related to damage to the same regions as total error detection, but the relationship was stronger, resulting in a larger cluster of significant lesion-deficit association (8,046 mm^3^), centered at −31.8, 9.4, 26.1 (Figure 3). The stronger lesion-behavior relationship observed for phonological error detection suggests that the localization of total error detection was driven primarily by phonological error detection. To confirm that the results did not simply reflect reduced speech output among individuals with frontal white matter damage, we added MLU as a covariate to our SVR-LSM analysis of phonological error detection. The result remained significant after controlling for fluency and centered more closely to the DLPFC. A single cluster was identified with a volume of 9,641 mm^3^, and center of mass at MNI coordinates −37.3, 9.3, 28.7 (Figure 4). No significant clusters were identified in the SVR-LSM analysis of semantic error detection (see Figure 1 in the online supporting information). These results did not change substantially when reanalyzed using error detection performance based only on the 60 PNT items administered to all participants (see the online supporting information).

### ROI Analyses for Semantic Error Detection

In light of the reduced sample size for the SVR-LSM analysis of semantic error detection, theory-driven ROIs were selected to determine the association of semantic error detection with lesions in specific brain regions involved in auditory comprehension, lexical processing, word production, and executive function (Table 5). The results of all ROI analyses were null, even at an uncorrected statistical threshold.

**Table 5.** Results of region of interest analyses

	Semantic error detection (*N* = 29)
Anterior superior temporal gyrus	0.035
Posterior superior temporal gyrus	0.298
Posterior middle temporal gyrus	0.153
Heschl’s gyrus	0.126
Planum temporale	0.208
Angular gyrus	0.297
Inferior frontal gyrus	−0.065
Middle frontal gyrus	−0.167

*Note*. Partial correlations between region of interest (ROI) lesion load and semantic error detection, controlling for total lesion volume. Negative values indicate a relationship between ROI damage and behavioral impairment. ***p < 0.05 after Bonferroni correction for 8 comparisons, **p < 0.01 uncorrected, *p < 0.05 uncorrected.

## DISCUSSION

In this study, we examined the behavioral and lesion correlates of impaired detection of speech errors in people with aphasia. We found that reduced detection of phonological errors was associated with low fluency and low performance on measures of executive control. Reduced semantic error detection related to low performance on a test of word-level comprehension ability. Finally, we found that damage to frontal white matter and the DLPFC was associated with poor error detection. Overall, these findings provide valuable evidence regarding the neural and behavioral underpinnings of speech error monitoring, and suggest that deficits in monitoring of phonological and semantic errors relate to somewhat different mental processes. Findings are consistent with aspects of both production- and comprehension-based models of error monitoring.

### Support for Production-Based Error Monitoring Models

Fluency marks successful speech production, and in this sense, an association between fluency and phonological error detection supports production-based monitors in general. A more precise account of this relationship is less clear. The conflict model of Nozari and colleagues (2011) predicts phonological error monitoring to decrease as a result of higher noise in the system responsible for the activation of phonological nodes. Low fluency could reflect increased noise in the phonological system causing delays in retrieval, but this interpretation is not consistent with our lesion-symptom mapping result, given that phonological error production has been associated with lesions of the parietal lobe (Mirman et al., 2015; Schwartz, Faseyitan, Kim, & Coslett, 2012). Alternatively, since both fluency and phonological errors are associated with deficits in postlexical speech production (Nickels & Howard, 1995; Romani, Olson, Semenza, & Granà, 2002; Schwartz, Wilshire, Gagnon, & Polansky, 2004; Wilshire, 2002), the association between fluency and detection of phonological errors may imply that these errors are detected during postlexical speech production. Although the Nozari conflict model does not include postlexical speech production stages, many production-based models of error monitoring suggest that monitoring also occurs at these stages (see Postma, 2000 for a review).

Our lesion-symptom mapping results are also consistent with models suggesting that error detection relies on speech production processes. Reduced error detection related to damage to a large region of frontal white matter underlying areas associated with speech production (Price, 2010). Prior studies have indicated that white matter tracts in this region are responsible for supporting fluent speech (Catani et al., 2013; Mandelli et al., 2014), which is consistent with our behavioral findings. A recent fMRI study in healthy participants also provided support for a speech monitor dependent on frontal regions. Gauvin and colleagues (2015) found that self-monitoring elicits activity across several areas involved in speech production, such as the presupplementary motor area (pre-SMA) and IFG (Gauvin et al., 2015). Our results add causal evidence that frontal white matter tracts are involved in successful detection of speech errors.

Recent work has implicated the left frontal aslant tract (FAT), which connects left IFG to pre-SMA and SMA, in the initiating, sequencing, and stopping of language production (Catani et al., 2013; Dick, Garic, Graziano, & Tremblay, 2018). Given that the white matter implicated in our study was adjacent to the IFG, it is logical to ask whether the FAT is involved in self-monitoring. However, a number of white matter tracts run through the areas implicated here, including tracts connecting medial and lateral frontal regions such as the FAT, tracts connecting subcortical structures and frontal regions, and tracts connecting frontal regions with temporoparietal areas. A future diffusion tensor imaging study could clarify the specific white matter connections that are essential for proper speech error monitoring.

### The Role of Executive Control in Speech Error Monitoring

Considerable debate centers on whether the monitor used to detect one’s own speech errors is domain-general or language-specific. The perceptual loop model predicts the monitor to be specific to language since it should depend on the speech comprehension system (Levelt, 1983; Postma, 2000). The conflict-based model of Nozari and colleagues (2011) is a domain-general model in that conflict signals arising during naming are received by a frontal brain structure (namely, the ACC), which monitors errors produced in other cognitive domains as well (Nozari et al., 2011). Domain-general theories of error monitoring predict successful detection to rely on executive functioning, regardless of the type of error being detected (Botvinick et al., 2001; Yeung et al., 2004). In support of this prediction, we identified a relationship between two measures of executive function (digit span difference and spatial span difference) and the monitoring of phonological errors. However, in contrast to the predictions of a domain-general theory, we did not find an association between these measures and semantic error detection. The finding that the monitoring of different types of errors relates to different behavioral measures may be interpreted as evidence against a domain-general model. However, it is also worth noting that we found a correlation between phonological error detection and semantic error detection, implying that some overlapping processes underlie monitoring of both error types. We also found some support for the anatomical predictions of a domain-general model for error monitoring, at least in the analysis of phonological errors. Reduced error detection related to damage to the DLPFC, a region known to be associated with working memory and domain-general executive function (Miller, 2001; Miller & Cohen, 2001).

Nozari and colleagues’ conflict-based model (2011) predicts the ACC to be involved in error monitoring, and prior evidence in healthy participants indicates that internal speech monitoring recruits ACC activity (Gauvin et al., 2015). Frontal white matter lesions may disrupt connectivity to the ACC, resulting in impaired error detection (Hogan, Vargha-Khadem, Saunders, Kirkham, & Baldeweg, 2006), but since we lacked lesion coverage of the ACC in our participant group, we cannot provide evidence for or against a causal role for this region in speech error monitoring. Future work using connectome-based lesion methods that can detect disconnected regions outside the lesioned area (Gleichgerrcht, Fridriksson, Rorden, & Bonilha, 2017; Yourganov, Fridriksson, Rorden, Gleichgerrcht, & Bonilha, 2016) will be useful to examine this issue further.

It is worth considering whether our tasks for executive control–that is, those designed to detect differences between forwards and backwards digit/spatial span–appropriately measure the type of control required to resolve conflict within the framework of conflict monitoring theory. Within the conflict monitoring framework, top-down control is required to override a habitual response (e.g., the habitual response to read the word instead of name the color in the Stroop task) and is provided by the DLPFC (MacDonald, Cohen, Stenger, & Carter, 2000; Yeung, 2015). This type of control is likely at play when completing the backwards digit or spatial span task, since the habitual response of repeating a sequence in the same order as it was presented must be overridden and replaced with a manipulation of that sequence in the reverse direction. Subtracting the forwards digit/spatial span score from the backwards score adjusts for the demands on maintenance in short-term memory required for both tasks, isolating the executive control component in the resulting measure. However, other factors could also contribute to a difference in performance on the forwards versus backwards digit/spatial span tasks. Some evidence indicates that separate mechanisms, one based on refreshing and another based on rehearsal, are responsible for maintaining short-term memory during low demand tasks like forwards digit span on the one hand and high demand tasks like backwards digit span on the other (Baddeley, 2003; Camos, Lagner, & Barrouillet, 2009; Camos, Mora, & Oberauer, 2011; Ghaleh et al., 2019). Therefore, a selective deficit in backwards but not forwards digit span could reflect an impairment in systems that support the articulatory rehearsal of phonological information (i.e., the phonological loop; Ghaleh et al., 2019), and it could be this aspect of the digit span difference score that is associated with poor error detection. The finding that the difference between backwards and forwards spatial span also related to phonological error detection makes this interpretation less likely, assuming that rehearsal for backwards spatial span relies on a visuospatial sketchpad rather than the phonological loop. Another area of less clarity is the null relationship between semantic error detection and measures of executive function. The null finding is inconsistent with a domain-general model, but it may simply reflect reduced power for the semantic error detection analyses. Alternatively, our behavioral measures could be less sensitive to the type of control necessary for manipulation of semantic information. Future work comparing error detection ability with performance on a wide range of executive function and working memory tasks could clarify which cognitive abilities are required to successfully monitor speech errors.

### Mechanisms of Semantic Error Detection

Our finding that the detection of semantic errors relies on word-level comprehension is consistent with comprehension-based models of monitoring that suggest that monitoring impairments follow an inability to comprehend one’s own speech (Levelt, 1983). While prior studies did not find a relationship between auditory comprehension and error monitoring (Marshall et al., 1998; Marshall et al., 1985; Nickels & Howard, 1995), those studies investigated phonological, not semantic errors. A role for comprehension in semantic error monitoring would account for evidence of poor semantic error detection among patients with severe comprehension deficits (Marshall et al., 1985).

However, some evidence suggests that both word comprehension and word production rely on the same lexical-semantic representations (Foygel, 2000; Hanley & Nickels, 2009). An alternative interpretation of the word-to-picture matching measure is as an indicator of lexical-semantic access ability, which is required for naming as well as comprehension (Hanley & Nickels, 2009). Consistent with this interpretation, word-picture matching scores and naming (PNT) scores correlated more strongly with each other than either did with any other measure examined in our participants (Table 4). Semantic errors occur primarily during the lexical-semantic stage of word production due to a failure to activate the correct lemma from semantics (Foygel, 2000; Schwartz et al., 2009). As noted above, according to the conflict-based model, damage to lexical-semantic access is expected to result in high levels of conflict that cause difficulty detecting semantic errors (Nozari et al., 2011). Thus, the relationship between word-picture matching and semantic error detection could be viewed as consistent with this model.

While lesion-behavior associations in the SVR-LSM or ROI analyses might have helped clarify the mechanisms by which semantic errors are monitored, the null findings of these analyses proved unhelpful. These analyses were limited by the small sample size available. Additional studies with larger populations and more specific behavioral measures will be needed to identify the neural and behavioral correlates of semantic error detection.

### Limitations

In order to capture the reflexive aspect of natural error detection, we did not give participants explicit instructions to comment on the accuracy of their naming attempts, consistent with past studies of self-monitoring in people with aphasia (Nozari et al., 2011; Schuchard et al., 2017; Schwartz et al., 2016). However, it is possible that some patients were aware of the errors they were making, but chose not to comment on them. While in theory this issue could be addressed using a paradigm in which participants are required to report their accuracy following each naming trial, this has the potential to alter the way they approach error monitoring. We also note that combining the two cohorts of patients was necessary to achieve an adequate sample size, and behavioral measures examined here were selected as the best measures available in both cohorts. Additional measures, for instance examining nonverbal semantics, speech perception, or motor speech production, would have been informative but were not available in both cohorts. Future prospective studies of error monitoring should select measures that allow a more precise and comprehensive delineation of the behavioral correlates of error detection.

### Conclusion

Gaining a more detailed understanding of the brain and behavioral basis of speech self-monitoring may lead to new treatments aimed at improving awareness of errors and self-correction in aphasia. These results demonstrate that monitoring of different error types relies on distinct cognitive functions, and provide causal evidence for the importance of frontal white matter tracts and DLPFC for self-monitoring of speech. These findings substantially inform the debate regarding the neural and behavioral underpinnings of speech error monitoring.

## FUNDING INFORMATION

Peter E. Turkeltaub, National Center for Advancing Translational Sciences (http://dx.doi.org/10.13039/100006108), Award ID: KL2TR000102. Peter E. Turkeltaub, Doris Duke Charitable Foundation (http://dx.doi.org/10.13039/100000862), Award ID: 2012062. Peter E. Turkeltaub, National Institute on Deafness and Other Communication Disorders (http://dx.doi.org/10.13039/100000055), Award ID: R03DC014310. Peter E. Turkeltaub, National Institute on Deafness and Other Communication Disorders (http://dx.doi.org/10.13039/100000055), Award ID: R01DC014960. Mackenzie E. Fama, National Institute on Deafness and Other Communication Disorders (http://dx.doi.org/10.13039/100000055), Award ID: F31DC014875. Andrew T. DeMarco, National Center for Advancing Translational Sciences (http://dx.doi.org/10.13039/100006108), Award ID: TL1TR001431.

## AUTHOR CONTRIBUTIONS

Ayan S. Mandal contributed to the study concept, acquisition, and analysis, and the interpretation of data, and drafted the manuscript. Mackenzie E. Fama contributed to the acquisition of data, and the study concept and design. Laura M. Skipper-Kallal contributed to the interpretation of data and edited the manuscript. Andrew T. DeMarco contributed to the analysis of data and edited the manuscript. Elizabeth H. Lacey contributed to the acquisition of data, and the study concept and design. Peter E. Turkeltaub contributed to the study concept and design, and the interpretation of data, and edited the manuscript. All authors reviewed and commented on the final version of the manuscript.
