# Supplementary figures and images for: Brain Structures and Cognitive Abilities Important for the Self-Monitoring of Speech Errors
Source: Neurobiol Lang (Camb). 2020 Aug 1;1(3):319–38. doi: 10.1162/nol_a_00015 (PMC8528269; doi:10.1162/nol_a_00015)

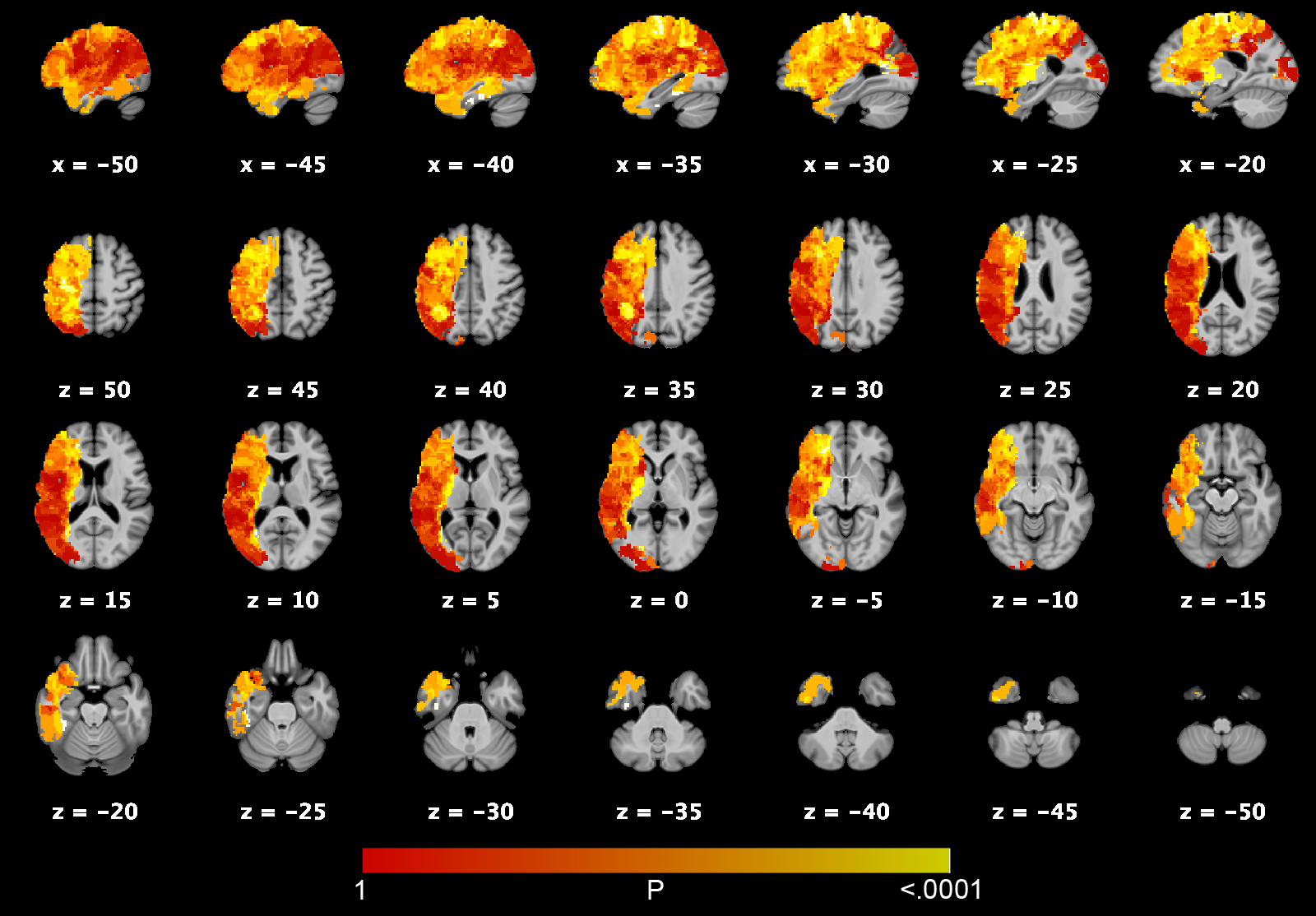

Supplement: Supplementary file 1 [file nol-1-3-319-s001.tiff]

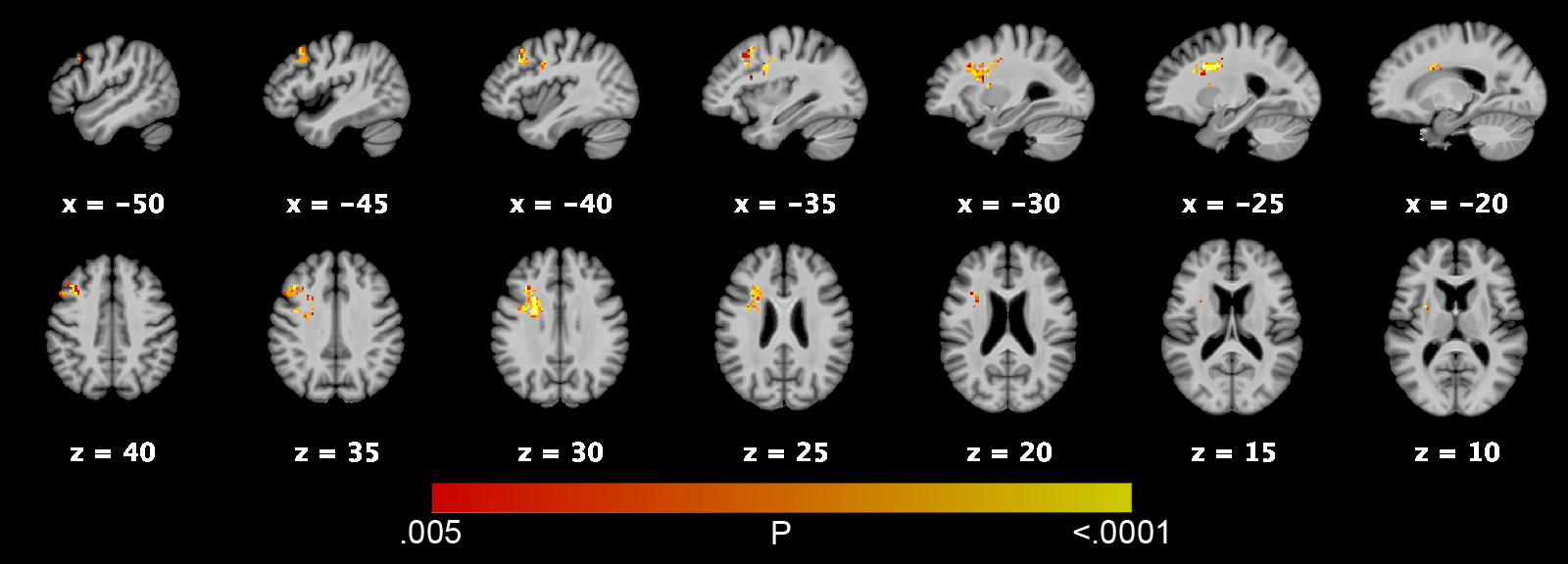

Supplement: Supplementary file 2 [file nol-1-3-319-s002.tiff]

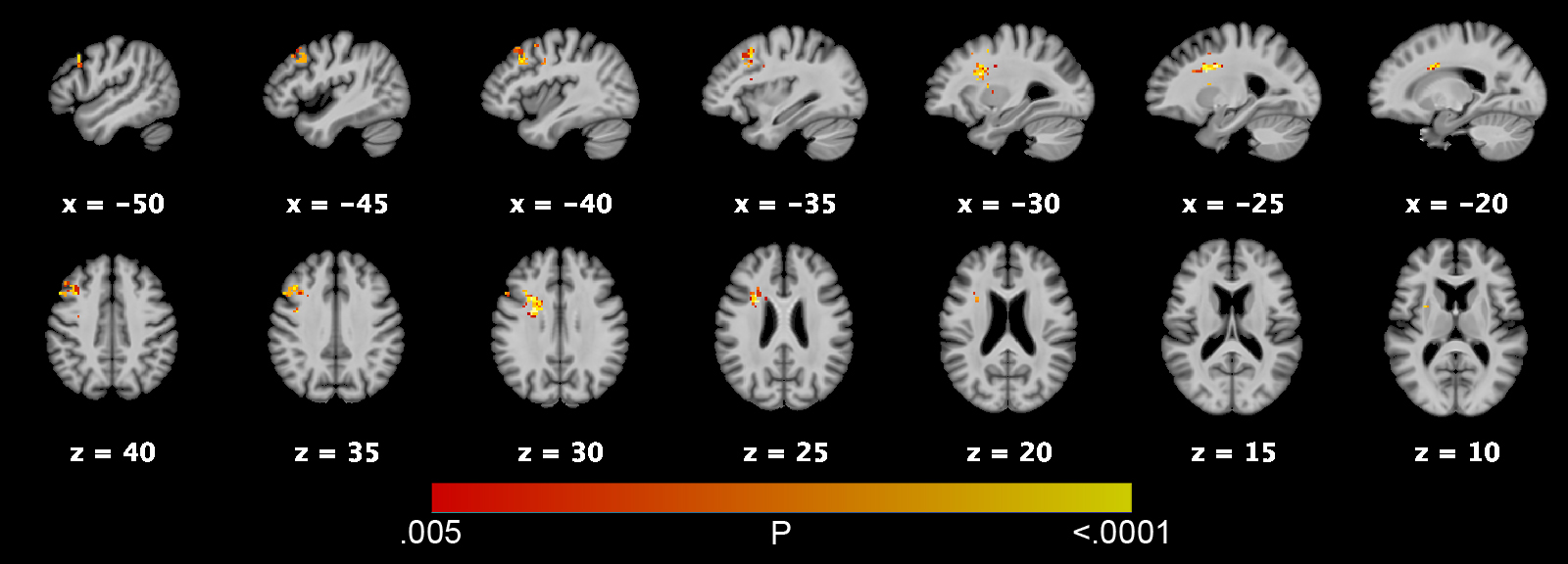

Supplement: Supplementary file 3 [file nol-1-3-319-s003.tiff]
